# Fibrous Extracellular Spheroids in an Endoscopic Ultrasound-Guided Pancreatic Fine Needle Aspiration Correlating to a Gyriform Pancreatic Endocrine Tumor with a Unique Cobblestone Pavement Growth Pattern

**DOI:** 10.1155/2019/1701072

**Published:** 2019-10-17

**Authors:** Alessandro Marotta, Jordan P. Reynolds, Thomas P. Plesec, E. Rene Rodriguez, Sunguk N. Jang, Maria Luisa C. Policarpio-Nicolas, Bridgette Springer, Charles D. Sturgis

**Affiliations:** ^1^Tomsich Pathology and Laboratory Medicine Institute, Cleveland Clinic, Cleveland, OH, USA; ^2^Gastroenterology, Cleveland Clinic, Cleveland, OH, USA

## Abstract

Pancreatic neuroendocrine neoplasms (PanNENs) are uncommon tumors. Fine needle aspiration (FNA) samples from PanNENs are typically of high cellularity and lack necrosis. In cytology slides from these tumors, dyscohesive cells are usually reported with variably round to oval to plasmacytoid forms exhibiting coarsely granular chromatin and showing immunoreactivity for synaptophysin. We present an unusual, and to our knowledge not previously described, example of an FNA of a PanNEN with large extracellular fibrous spheroids containing intrinsic fibroblasts and rimmed by small to intermediate sized neoplastic epithelial cells with high nuclear cytoplasmic ratios. The cytomorphology of the PanNEN in this case was in some ways reminiscent of that expected in adenoid cystic carcinomas of the salivary glands that most often contain large extracellular globules of basement membrane material and a somewhat biphasic population of lesional cells. The cytomorphology in this case was found to correlate well with the resection specimen histomorphology of an exaggerated gyriform pattern of growth resulting in a unique cobblestone-pavement like microscopic appearance. Knowledge of this potential cytomorphology will aid the cytology community through recognition and reporting of this previously undescribed pattern in an uncommon disease.

## 1. Introduction

Pancreatic neuroendocrine neoplasms (PanNENs) are uncommon lesions that occur in approximately 1 out of 100,000 people and constitute 2% of all pancreatic tumors [1–3]. These lesions are the second most common form of primary pancreatic neoplasia, after pancreatic ductal adenocarcinomas [1, 4]. Neuroendocrine neoplasms of the pancreas most often present sporadically in older adults, between the sixth and eighth decades of life; however, PanNENs are sometimes reported in younger patients, especially in association with multiple endocrine neoplasia (MEN) and Von Hippel Lindau (VHL) syndromes [5]. Within the last 4 decades, the incidence of PanNENs has gradually increased, as smaller, non-functional and otherwise asymptomatic tumors have been more readily detected by imaging [2, 4]. In recent years, the percentage of diagnosed non-functional (non-hormone secreting) PanNENs has risen to 80%. These lesions may appear as either solid or cystic masses by imaging [5].

Advances in endoscopic ultrasound-guided fine-needle aspiration (EUS-FNA) have been a boon to the detection, diagnosis and staging of PanNENs [2, 5]. EUS-FNA has emerged as the most sensitive modality for detecting and diagnosing pancreatic mass lesions, especially those that are 2 cm or less in greatest dimension [4, 5]. When compared with computed tomography (CT), EUS has a superior sensitivity for detecting small pancreatic tumors, EUS 92% sensitive vs CT 63% sensitive [5]. Despite higher sensitivity, EUS-FNA for a specific diagnoses of a PanNEN may in some cases be challenging. Accuracy of diagnosis is increased when procedures are performed by experienced endoscopists working closely with cytopathologists at the time of rapid on site evaluation to ensure optimal sampling and specimen triage [2].

We present the cytology of an EUS-FNA sampled PanNEN with a unique, and to our knowledge, previously unreported cytomorphologic appearance of prominent extracellular spheroids of intrinsic fibrous tissue. The cytology correlates to the histoarchitectural appearance of a gyriform type PanNEN with an unusual and exaggerated fibrous cobblestone-pattern of growth. The final resection specimen and ancillary studies in this case confirm the diagnosis of a PanNEN and exclude the key morphologic differential diagnosis of a pancreatic solid-pseudopapillary neoplasm (PSPPN).

## 2. Case Report

**Figure 1:**
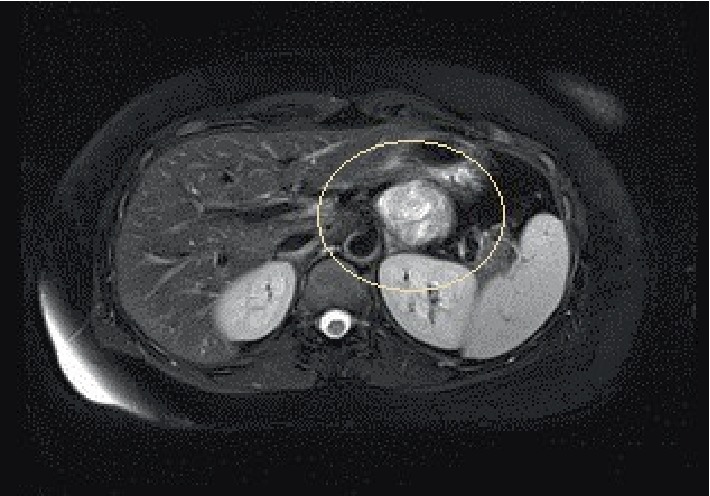
Magnetic resonance imaging (MRI) confirmed a 4.6 cm relatively well circumscribed, hypervascular, enhancing mass (circled) in the distal pancreatic body with no associated pancreatic ductal dilatation.

**Figure 2:**
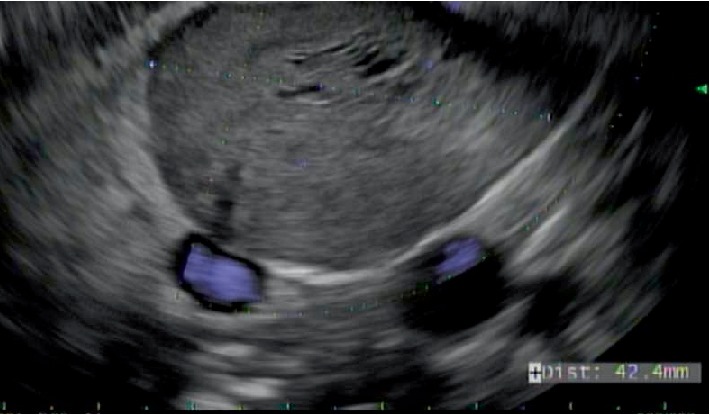
Endoscopic Ultrasound (EUS) examination demonstrated an oval, hypoechoic mass with well-defined borders and no evidence of associated regional lymphadenopathy. No invasion of regional vasculature was present.

The patient was a 41-year-old female with a past medical history significant for an anxiety disorder, clinical depression and obesity. She was a lifelong never smoker and reported occasional use of alcohol. She had no personal or first degree family member history of visceral malignancy. She presented approximately one month prior with complaints of dull, achy, right upper quadrant abdominal pain. This pain worsened after eating. A low fat diet was prescribed with incomplete resolution of symptoms. Esophagogastroduodenoscopy (EGD) was performed and showed no focal findings. Right upper quadrant ultrasonography (US) and magnetic resonance imaging (MRI) studies confirmed a 4.6 × 4.2 × 3.5 cm mass lesion in the distal pancreatic body with no pancreatic duct dilatation (Figure 1). Because of concerns for neoplasia/malignancy, the patient was referred for EUS evaluation. This confirmed a greater than 40 mm oval mass lesion with well-defined endosonographic borders in the distal pancreatic body (Figure 2). Vessels, including splenic vein, splenic artery and superior mesenteric artery, showed no sign of involvement, with the mass appearing to compress the aforementioned vessels without direct abutment. Four transgastric fine needle aspiration passes were performed using a 22 gauge needle (SNJ). Adequate cellularity was confirmed by rapid on-site evaluation (ROSE) (JPR). No adverse effects of the procedure were recorded.

**Figure 3:**
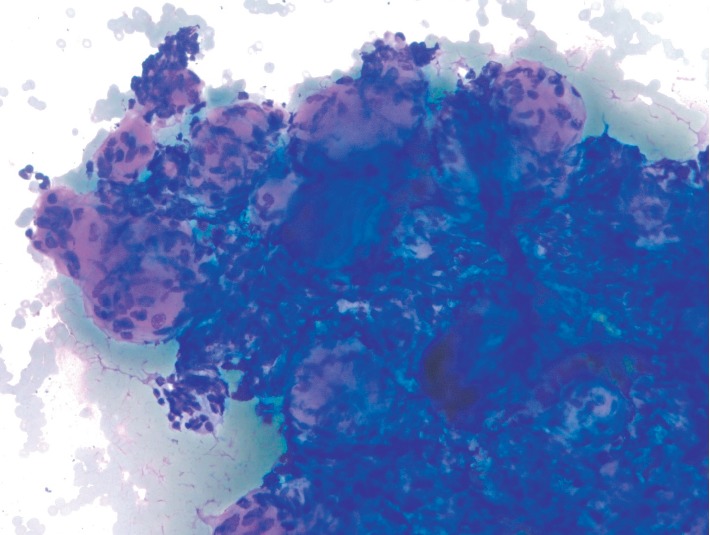
Three-dimensional microbiopsy tissue particles predominated. These show homogenous magenta spheroids with intimately associated spindled cells and collections of intervening, high nuclear/cytoplasmic ratio, small to intermediated sized cells with crush artifact (Modified Giemsa stain, 200X).

**Figure 4:**
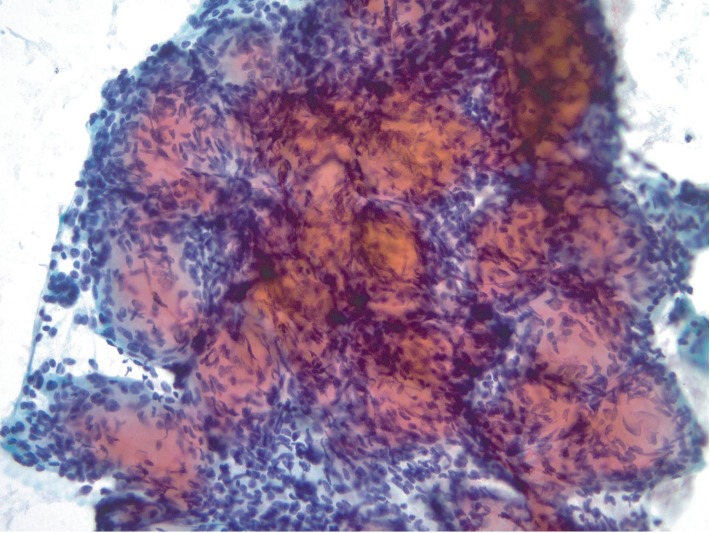
Large, cohesive, three-dimensional aggregates of cellular material predominate in the direct smears. These show spheroidal globules of eosinophilic to amphophilic extracellular material with intimately associated small to intermediate sized spindled and epithelioid cells. While appliqueed spindled cells are noted on the spheroids, true intrinsic fibrovascular cores are not identified (Papanicolaou stain, 200X).

**Figure 5:**
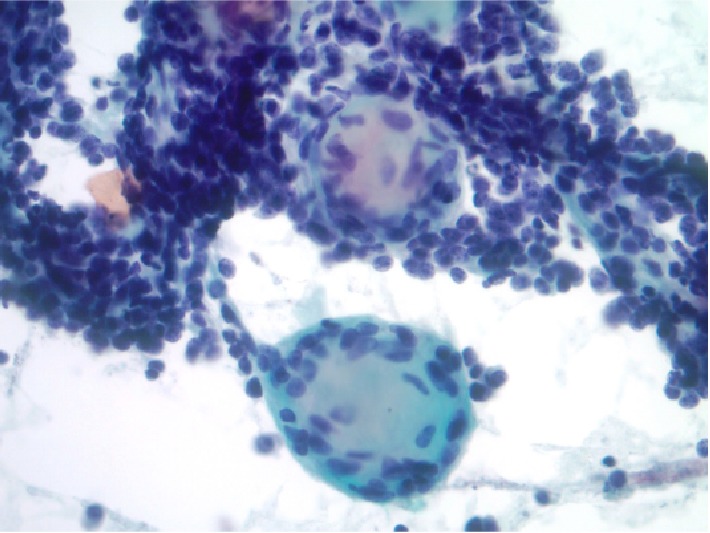
Cyanophilic homogenous spheroid with adherent or intrinsic bland spindled cells (fibroblasts) and collections of small, high nuclear/cytoplasmic ratio round to oval smears intervening between (Papanicolaou stain, 400X).

**Figure 6:**
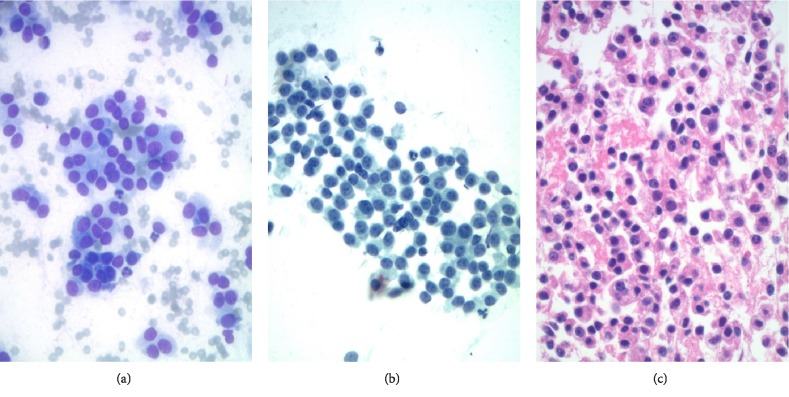
(a) Scattered loosely cohesive collections of epithelioid cells were noted in the direct smear backgrounds (Modified Giemsa stain, 400X). (b) These cells varied in shape from round to oval to plasmacytoid with finely granular chromatin and occasional minute nucleoli (Papanicolaou stain, 400X). (c) These cells appeared similar to the cells between the globules in the cell block but were dyscohesive (Hematoxylin & eosin stain, 400X).

**Figure 7:**
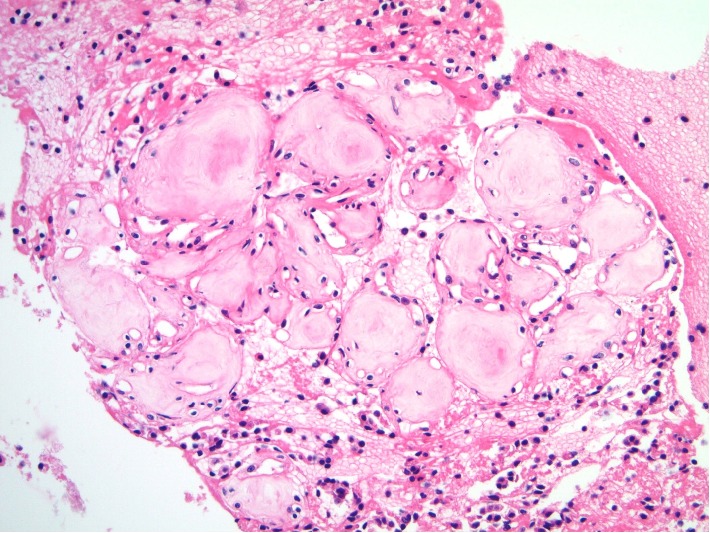
The cell block showed loose collections of small epithelioid cells and in some areas connected disc-like spheres of hyalinized fibrous tissue with adherent/intrinsic bland spindle cells, suggesting a possible papillary/arborizing morphology (Hematoxylin & eosin stain, 200X).

**Figure 8:**
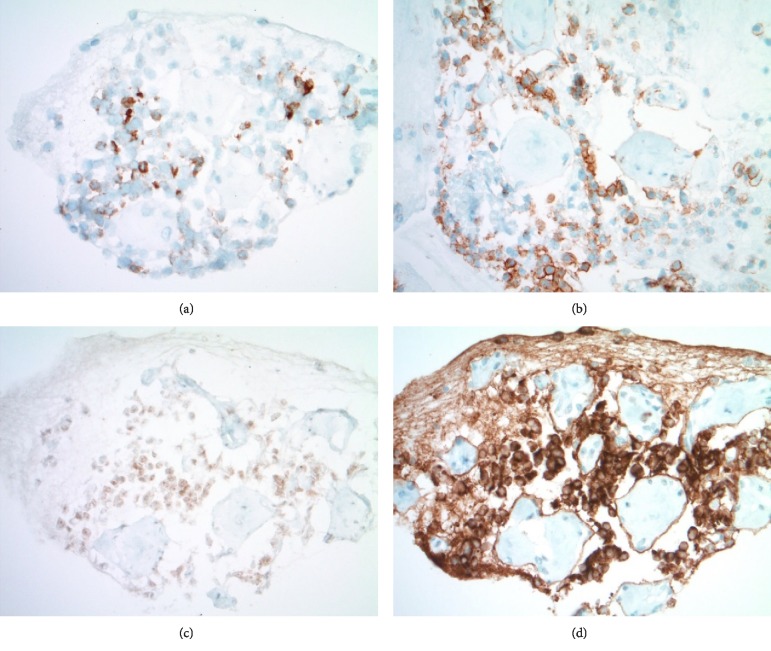
(a) Cell block showing cytoplasmic immunoreactivity for scattered small to intermediate sized, high nuclear/cytoplasmic ratio cells present between spheroids (CK AE1-3 immunohistochemistry, 400X). (b) Cell block with similar pattern of immunoreactivity (*β*-catenin immunohistochemistry, 400X). (c) Cell block with patchy/weak cytoplasmic immunoreactivity (Chromogranin A immunohistochemistry, 400X). (d) Cell Block with diffuse and strong immunoreactivity in epithelioid cells, while spheroids and intrinsic spindled nuclei appear nonreactive (Synaptophysin immunohistochemistry, 400X).

The cytology specimen consisted of eight direct smears (four air-dried/modified Giemsa stained slides and four alcohol-fixed/Papanicolaou stained slides) as well as 30 ml of blood-tinged CytoLyt (Hologic) fluid containing particles. The fluid portion of the specimen was used to create a ThinPrep (Hologic) liquid based slide, and a Cellient (Hologic) cell block was also created. The modified Giemsa stained slides were used for ROSE with interpretations of adequate cellularity and atypical cells given. The majority of the cellular material in the ROSE slides consisted of three-dimensional microbiopsy tissue particles which were present predominantly in the central areas of the direct smears. These microbiopsy particles contained homogenous magenta spheroids with intimately associated spindled cells and collections of intervening, high nuclear/cytoplasmic ratio, small to intermediated sized cells with crush artifact (Figure 3). Similar appearing three-dimensional tissue particles were later seen on the alcohol-fixed smears. In the Papanicolaou stained slides, these particles contained dense spheroidal globules of eosinophilic to amphophilic extracellular material with intimately associated small spindled cells and adjacent round to oval epithelioid cells. While appliqueed spindled cells were noted on the globules, true intrinsic fibrovascular cores were not identified (Figure 4). At higher magnification in less complex cell groups, individual globular spheroids appeared cyanophilic in the Papanicolaou stained slides, and these structures showed either adherent or intrinsic small bland spindled nuclei (Figure 5). In most of the slides, smaller numbers of loosely cohesive to dyscohesive epithelioid cells were present. These cells had minimal cytoplasm, were small to intermediate in size and varied from round to oval to occasionally plasmacytoid (Figure 6). No background necrosis was present. The cell block showed similar findings with eosinophilic spheroidal structures. In some areas of the cell block the spheroids were minimally associated with the small epithelioid cells and appeared as hyalinized discs with intimately associated bland spindled cells, possibly interpreted as a papillary architectural pattern (Figure 7). Ancillary immunohistochemical tests were performed on sections from the cell block. These showed immunoreactivity with cytokeratin AE1-3, chromogranin, synaptophysin and *β*-catenin (Figure 8). A diagnosis of neoplasia, favor a PanNEN was given with a comment stating that a pancreatic solid pseudopapillary neoplasm could not be entirely excluded.

**Figure 9:**
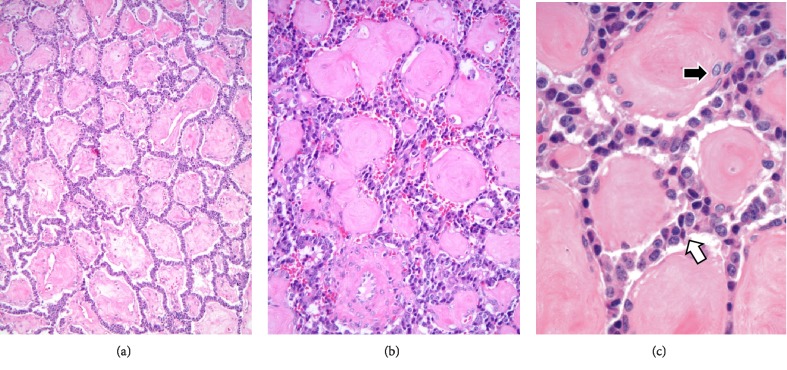
(a) Histologic sections from the pancreatectomy specimen confirmed a solid neoplasm with a low power pattern reminiscent of pulmonary alveolar edema or proteinosis with peripheral rims of epithelial cells surrounding eosinophilic spaces (Hematoxylin & eosin, 100X). (b) At greater magnification, the collagenized nature of the eosinophilic cobblestones is apparent with lesional epithelial cells in ribbons between the spheroids (Hematoxylin & eosin, 200X). (c) At highest magnification, the small, bland, tapering and spindled nuclei (black arrow) within the spheroids can be readily distinguished from the lesional epithelial cells between the cobblestones (white arrow) (Hematoxylin & eosin, 600X).

**Figure 10:**
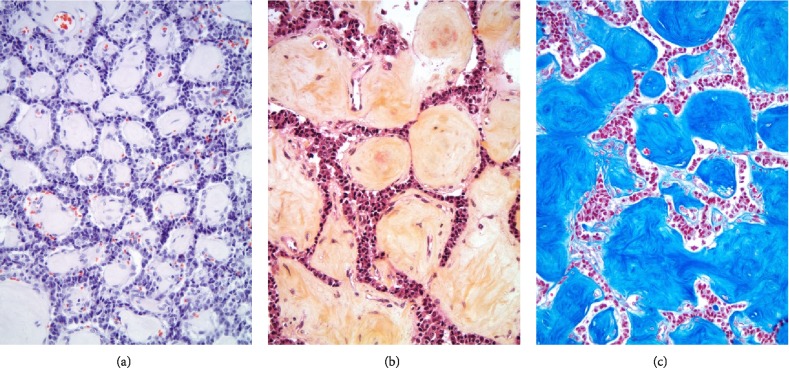
(a) Absence of brick red staining and no evidence of apple-green birefringence on polarized light excludes amyloid protein deposition in the cobblestones (Congo red stain, 200X). (b) Orange-pink staining with intrinsic fibrils in sections of the cobblestones supports the impression of spheroids of fibrous tissue (Movat stain, 200X). (c) Azure blue staining with intrinsic fibrils further indicates that the homogenous and paucicellular spheroids are comprised of fibrous tissue (Trichrome stain, 200X).

**Figure 11:**
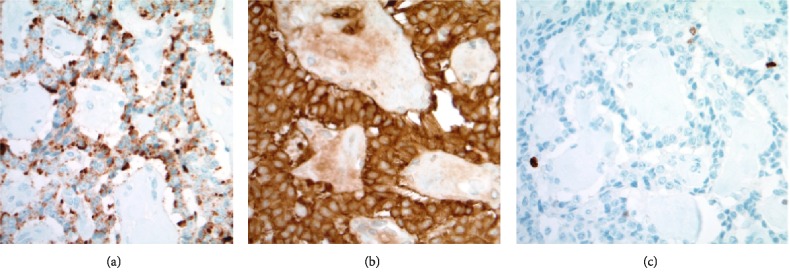
(a) Diffuse granular cytoplasmic immunoreactivity is present in the ribbons of lesional epithelial cells between fibrous spheroids (Chromogranin A immunohistochemistry, 400X). (b) Diffuse and strong homogenous cytoplasmic immunoreactivity is confirmed in the epithelial cells (Synaptophysin immunohistochemistry, 400X). (c) Very rare epithelial nuclei (<3% of lesional cells) show immunoreactivity with the proliferation marker (Ki-67 immunohistochemistry, 400X).

Six weeks after the EUS-FNA procedure, the patient underwent distal pancreatectomy. Surgical resection confirmed a pancreatic neuroendocrine tumor (PanNET), grade 1 with negative margins and four uninvolved regional lymph nodes. The majority of the histologic sections showed an exaggerated gyriform pattern of growth resulting in a cobblestone pavement-like architectural pattern with eosinophilic discs of fibrous stroma surrounded by neoplastic cells (Figure 9). The neoplastic cells showed features typical of a neuroendocrine tumor on histology with regular, rounded nuclei and stippled chromatin. Cytochemical stains including Congo red, Movat and Trichrome stains were performed. These helped to exclude amyloid deposition and to confirm that the spheroids were truly comprised of fibrous tissue (Figure 10). Polarized light examination further supported the conclusion that the extracellular eosinophilic spheroids were comprised of collagen. Ancillary immunohistochemical studies undertaken on sections from the resected tumor confirmed diffuse immunoreactivity with chromogranin and synaptophysin with a nonreactive result for *β*-catenin and with a low (<3%) proliferation index on Ki-67 testing (Figure 11). Six months after distal pancreatectomy, the patient is well and is being treated with careful observation, with no evidence of disease recurrence or metastasis.

## 3. Discussion

Gastroenteropancreatic neuroendocrine neoplasms occur infrequently, but their incidence has increased six-fold over the last three decades [6]. Reported risk factors for PanNENs include tissue injury, germline predisposition, and others. In the United States, neuroendocrine neoplasms of the rectum and small intestine have the highest incidence with more cases reported than for neuroendocrine neoplasms of the pancreas and other gastrointestinal sites [6]. Increases in detection rates of asymptomatic pancreatic tumors account in part for the associated increases in incidence of neuroendocrine tumors. Recent scholarly publications have emphasized updates to the criteria for diagnosis, classification, and grading of PanNENs [1, 4, 7]. The 2017 WHO Classification divides PanNENs into malignant but well-differentiated pancreatic neuroendocrine tumors (PanNETs) as well as malignant poorly-differentiated neuroendocrine carcinomas (PanNECs). PanNETs are further subdivided into grade 1 (low grade), grade 2 (intermediate grade) and grade 3 (high grade) disease processes based upon morphology, mitotic indices and Ki-67 proliferation indices.

PanNETs are known to have varied histo-architectural and cytologic appearances with the histomorphology of well-differentiated PanNETs being similar to that of low grade neuroendocrine tumors at other body sites. PanNETs are often delineated from background pancreatic parenchyma by a pushing border and sometimes a fibrous pseudocapsule. Neoplastic cells can be arranged in many patterns with pseudoglandular, trabecular, organoidal, festooned, ribbon-like, angiomatoid, nested, rosette-like, and sheet-like patterns reported [4]. Gyriform growth with irregularly shaped island of fibrous tissue surrounded by thin ribbons of investing neoplastic epithelium is sometimes seen [8]. Varying degrees of stromal hyalinization have been reported in PanNETs, and it may be challenging to distinguish fibrous tissue from amyloid protein deposition in some cases [1, 4, 8]. The exaggerated cobblestone-appearing histomorphology in the current case represents an unusual example of a gyriform pattern of growth (Figure 9) with the large, extracellular, spheroidal globules in the cytology (Figure 5) correlating to the fibrous disc-like cobblestones in the histology. The appliqued bland spindle cells on/ in the fibrous spheroids in the cytology correlate to intrinsic fibroblasts in the cytology with the epithelial cells located between and around the spheroidal globules correlating to the neoplastic epithelium in the histology. Cytological evaluation of FNA slides of nonfunctioning PanNETs most often confirms high cellularity, clean (non-necrotic) backgrounds and loosely cohesive, round to oval (sometimes plasmacytoid) repetitive cells with coarsely granular chromatin [1]. Importantly, rare cytologic variants of this rare tumor type including oncocytic variants, rhabdoid tumors, vacuolated examples, lipid-rich forms, and clear cell morphologies are known to exist [1, 9–12].

Differential diagnostic considerations in both histopathologic and cytopathologic specimens may include adenocarcinomas, pancreatic solid pseudopapillary neoplasms (PSPPNs), acinar cell tumors, hematolymphoid neoplasia, chronic pancreatitis, metastatic neoplasms from distant body sites, and others. Cytologic overlap between cases of PanNETs and PSPPNs can lead to difficulty and challenge in accurately separating these entities. Some authors have emphasized morphologic nuances that may be of value in distinguishing PanNETs from a PSPPN. With clear cells, nuclear folds/grooves and small, intracellular hyaline globules favoring PSPPN [13, 14]. The 2017 World Health Organization (WHO) Classification emphasizes that PanNENs have significant expression of synaptophysin and also usually chromogranin A [1]. In individual cases with morphologic overlap, immunohistochemical (IHC) characterization may be of paramount importance to establishing an accurate diagnosis. Membranous E-cadherin immunoreactivity in PanNETs and nuclear *β*-catenin immunoreactivity in PSPPNs have been touted as sensitive and specific markers for the two tumors [15, 16]. In the FNA from our case, the cell block IHC for B-catenin showed patchy cell membrane and cytoplasmic staining (non nuclear staining) that in retrospect was spurious and was not seen in the resection specimen. Other IHC studies that may be of value in separating PanNETs from PSPPNs include but are not necessarily limited to CD56, INSM1, CD10, progesterone receptor and SOX-11 [16–18].

Ki-67 labeling has emerged as a cornerstone for histologic grading of PanNETs and is now incorporated into the WHO classification scheme [1]. In our center, Ki-67 proliferation indices are not routinely measured on FNA specimens; however, a growing literature on this topic does exist, with reports of good intraobserver agreement between cytology and histology cases [19–21]. It should be noted that Ki-67 labeling of EUS-FNA specimens should be carefully applied in clinical practice because of the possibility of grading underestimations in grade 2 to 3 PanNETs [21]. Assessment of proliferation indices in cytology specimens are likely of greatest value at predicting clinical behavior and assessing survival in patients who will not undergo surgical excision (because of comorbidities or because of the presence of metastases). Patients in these clinical settings may benefit from Ki-67 assessments on cytology specimens [22–23]. Ki-67 IHC was not pursued in our cytology case but was found to be low (<3%) in the resection specimen. It should be noted that randomly scattered pleomorphic cells (endocrine atypia) may be present in EUS-FNA samples from PanNETs, and pleomorphic and multinucleated cell have also been described in PSPPNs [24].

## 4. Conclusion

Overlap of cytomorphologic features between PanNETs and PSPPNs can result in difficulty when attempting to accurately classify EUS FNA samples, and in some instances ancillary IHC studies may not allow for exact classification, as the synaptophysin immunoreactivity expected in PanNETs may on occasion be present in PSPPNs, and spurious *β*-catenin immunoreactivity may be rarely encountered in PanNETs. A recent error reduction focused study of the cytologic criteria for the diagnosis of PSPPNs by EUS-FNA emphasized the presence of fine (and not coarsely granular) chromatin, nuclear grooves, pseudo papillae, pink stroma and small intracellular cytoplasmic hyaline globules as being statistically significantly associated with PSPPNs [24]. In our case of an FNA of a gyriform PanNET with an exaggerated cobblestone-like pattern of growth, a pseudopapillary pattern was considered because of the stromal spheroids with intrinsic fibroblasts, and in some areas pink stroma was noted. This case report details the unique cytomorphology of fibrous extracellular spheroids in FNA slides from a PanNET with a morphology that was reminiscent of the extracellular basement membrane material sometimes seen in FNAs of adenoid cystic carcinoma of the salivary glands and other body sites. Recognition that this unusual morphology is possible in PanNETs and that it correlates to an exaggerated variant of gyriform histologic growth may allow for accurate classification of similar tumors in the future.
